# Supplementary material for: From sequence to enzyme mechanism using multi-label machine learning
Source: BMC Bioinformatics. 2014 May 19;15:150. doi: 10.1186/1471-2105-15-150 (PMC4229970; doi:10.1186/1471-2105-15-150)
Supplement: Additional file 2 — Java code of ml2db. Additional file ml2db_code.tar.gz contains the Java source code to run the multi-label machine learning experiments and save the results to database. The code’s Javadoc is included. [file 1471-2105-15-150-S2.zip › additional file 2/ml2db/utils/doc/edu/cornell/lassp/houle/RngPack/RandomJava.html]

RandomJava


JavaScript is disabled on your browser.


- Overview
- Package
- Class
- Use
- Tree
- Deprecated
- Index
- Help

- Prev Class
- Next Class

- Frames
- No Frames

- All Classes

- Summary:
- Nested |
- Field |
- Constr |
- Method

- Detail:
- Field |
- Constr |
- Method


edu.cornell.lassp.houle.RngPack

## Class RandomJava

- java.lang.Object
- - cern.colt.PersistentObject
  - - edu.cornell.lassp.houle.RngPack.RandomElement
    - - edu.cornell.lassp.houle.RngPack.RandomJava

- All Implemented Interfaces:
  :   java.io.Serializable, java.lang.Cloneable

  ---

    

  ```
  public class RandomJava
  extends RandomElement
  ```

  RandomJava is a class wrapper for the `Math.random()` generator
  that comes with Java. I know nothing about the quality of
  `Math.random()`, but I will warn the reader that system-supplied
  RNGs have a bad reputation; RandomJava is **NOT** reccomended for
  general use, it has only been included as a straightforward example of how to
  build a `RandomElement` wrapper for an existing RNG. The
  RANMAR, RANECU and RANLUX generators included in
  this package all appear to be faster than Math.random(); all three are
  well-studied, portable and proven in use.

  Source code  is available.

  Version:
  :   1.0

  Author:
  :   Paul Houle  (E-mail: houle@msc.cornell.edu)

  See Also:
  :   `Ranmar`,
      `Ranlux`,
      `Ranecu`,
      Serialized Form

- - ### Constructor Summary

    Constructors

    Constructor and Description

    `RandomJava()`
  - ### Method Summary

    Methods

    | Modifier and Type | Method and Description |
    | --- | --- |
    | `double` | `raw()` Wrapper for `Math.random().` |

    - ### Methods inherited from class edu.cornell.lassp.houle.RngPack.RandomElement

      `choose, choose, gaussian, gaussian, powlaw, raw, raw, uniform`
    - ### Methods inherited from class cern.colt.PersistentObject

      `clone`
    - ### Methods inherited from class java.lang.Object

      `equals, getClass, hashCode, notify, notifyAll, toString, wait, wait, wait`

- - ### Constructor Detail


    - #### RandomJava

      ```
      public RandomJava()
      ```
  - ### Method Detail


    - #### raw

      ```
      public double raw()
      ```

      Wrapper for `Math.random().`

      **Specified by:**
      :   `raw` in class `RandomElement`

      Returns:
      :   a random double in the range (0,1).

      See Also:
      :   `RandomElement.raw()`


- Overview
- Package
- Class
- Use
- Tree
- Deprecated
- Index
- Help

- Prev Class
- Next Class

- Frames
- No Frames

- All Classes

- Summary:
- Nested |
- Field |
- Constr |
- Method

- Detail:
- Field |
- Constr |
- Method
